# Emerging Therapeutic Targets for Acute Coronary Syndromes: Novel Advancements and Future Directions

**DOI:** 10.3390/biomedicines12081670

**Published:** 2024-07-26

**Authors:** Andreas Mitsis, Michael Myrianthefs, Stefanos Sokratous, Georgia Karmioti, Michaela Kyriakou, Michail Drakomathioulakis, Stergios Tzikas, Nikolaos P. E. Kadoglou, Efstratios Karagiannidis, Athina Nasoufidou, Nikolaos Fragakis, Antonios Ziakas, George Kassimis

**Affiliations:** 1 Cardiology Department, Nicosia General Hospital, State Health Services Organization, Nicosia 2029, Cyprus; myr.michael@shso.org.cy (M.M.); stefanossokratous94@gmail.com (S.S.); georgiakarm@outlook.com (G.K.); michaelakyriakou95@yahoo.com (M.K.); bageragr@gmail.com (M.D.); 2 Third Department of Cardiology, Aristotle University of Thessaloniki, 54636 Thessaloniki, Greece; 3 Medical School, University of Cyprus, Nicosia 2115, Cyprus; kadoglou.nikolaos@ucy.ac.cy; 4 Second Department of Cardiology, Aristotle University of Thessaloniki, 54642 Thessaloniki, Greece; stratoskarag@gmail.com (E.K.); athinanassi@gmail.com (A.N.); fragakis.nikos@gmail.com (N.F.); gksup@yahoo.gr (G.K.); 5 First Department of Cardiology, AHEPA University Hospital, Aristotle University of Thessaloniki, 54636 Thessaloniki, Greece; aziakas@auth.gr

**Keywords:** acute coronary syndrome, antiplatelet agents, anti-inflammatory drugs, cardiovascular biomarkers, cardiovascular inflammation, clinical trials, plaque stabilization

## Abstract

Acute coronary syndrome (ACS) remains a major cause of morbidity and mortality worldwide, requiring ongoing efforts to identify novel therapeutic targets to improve patient outcomes. This manuscript reviews promising therapeutic targets for ACS identified through preclinical research, including novel antiplatelet agents, anti-inflammatory drugs, and agents targeting plaque stabilization. Preclinical studies have expounded these agents’ efficacy and safety profiles in mitigating key pathophysiological processes underlying ACS, such as platelet activation, inflammation, and plaque instability. Furthermore, ongoing clinical trials are evaluating the efficacy and safety of these agents in ACS patients, with potential implications for optimizing ACS management. Challenges associated with translating preclinical findings into clinical practice, including patient heterogeneity and trial design considerations, are also discussed. Overall, the exploration of emerging therapeutic targets offers promising avenues for advancing ACS treatment strategies and improving patient outcomes.

## 1. Introduction

Acute coronary syndrome (ACS) covers a relatively wide spectrum of clinical conditions, including unstable angina (UA), non-ST-segment elevation myocardial infarction (NSTEMI), and ST-segment elevation myocardial infarction (STEMI), characterized by acute coronary artery thrombosis and myocardial ischemia [1]. Despite advances in diagnosis and treatment, ACS remains a leading cause of morbidity and mortality worldwide, highlighting the need for continuous innovation in therapeutic approaches [2].

Current treatment strategies for ACS primarily focus on prompt restoring of coronary blood flow, minimizing myocardial damage, and reducing the risk of recurrent cardiovascular events. These strategies typically involve a combination of pharmacotherapy, percutaneous coronary intervention (PCI), and coronary artery bypass grafting (CABG) [3]. However, significant gaps in our understanding of ACS pathophysiology persist, and existing therapeutic options may not fully address the underlying mechanisms driving disease progression and adverse outcomes.

In recent years, there has been growing interest in exploring novel therapeutic targets for ACS, guided by insights from basic science research interpreting the molecular and cellular mechanisms underlying plaque instability, thrombosis, and subsequent myocardial injury [4]. Preclinical studies have identified promising targets for intervention, including novel antiplatelet agents, anti-inflammatory drugs, and agents targeting plaque stabilization pathways. This manuscript aims to review the latest advances in ACS therapeutics, focusing on emerging therapeutic targets identified through clinical and preclinical research and ongoing efforts to optimize ACS management and reduce in this area the global burden of cardiovascular disease.

## 2. Antiplatelet Agents

Platelet adhesion, activation, and aggregation play a central role in the pathophysiology of ACS, contributing to the formation of coronary artery thrombosis and subsequent myocardial ischemia [5]. Platelet activation is a complex process initiated by various stimuli, including endothelial injury, exposure to atherosclerotic plaque components, and release of inflammatory mediators [6]. Upon activation, platelets undergo a series of biochemical and morphological changes, leading to their recruitment to the site of vascular injury and formation of platelet-rich thrombi [7]. Following endothelial injury, circulating platelets adhere to the exposed subendothelial matrix through interactions between glycoprotein (GP) Ib receptors on platelets and von Willebrand factor (vWF) bound to collagen. Then the adherent platelets become activated in response to various agonists, including thrombin, adenosine diphosphate (ADP), and thromboxane A2 (TXA2) [8]. This activation process involves intracellular signalling pathways, resulting in increased expression of surface receptors, such as glycoprotein IIb/IIIa (GPIIb/IIIa) and P-selectin, and secretion of granule contents containing potent platelet agonists. Finally, the activated platelets undergo aggregation, mediated by binding of fibrinogen and vWF to activated GPIIb/IIIa receptors, leading to the formation of platelet aggregates and the initial formation of a platelet-rich thrombus [9].

In the setting of ACS, disruption of vulnerable atherosclerotic plaques exposes thrombogenic components, such as tissue factor, collagen, and lipid-rich debris, leading to platelet activation and aggregation. This process culminates in the formation of intracoronary thrombi, resulting in partial or complete occlusion of the coronary artery lumen and subsequent myocardial ischemia. The role of platelet activation and aggregation in ACS pathophysiology underscores the importance of antiplatelet therapy in ACS management [10]. Dual antiplatelet therapy (DAPT), typically consisting of aspirin and a P2Y12 receptor inhibitor (e.g., clopidogrel, ticagrelor, prasugrel), is a cornerstone of ACS treatment, aimed at inhibiting platelet activation and aggregation and preventing recurrent thrombotic events [11].

Clopidogrel is a prodrug that requires metabolic activation to exert its antiplatelet effects. It irreversibly inhibits the P2Y12 receptor, leading to reduced platelet aggregation. Clopidogrel has been extensively used in the management of ACS and in patients undergoing percutaneous coronary interventions (PCI) [12]. However, its efficacy can be variable due to genetic polymorphisms in the cytochrome P450 enzymes responsible for its activation, resulting in different levels of platelet inhibition among patients [13]. Prasugrel, another irreversible P2Y12 inhibitor, is more potent and has a faster onset of action compared to clopidogrel. Like clopidogrel, prasugrel requires metabolic activation, but it is less affected by genetic variability, providing more consistent antiplatelet effects [14,15]. Prasugrel has shown superior efficacy in reducing thrombotic events in patients with ACS undergoing PCI but is associated with a higher risk of major bleeding compared to clopidogrel [15]. Ticagrelor is a reversible, direct-acting P2Y12 inhibitor that does not require metabolic activation. It provides more consistent and potent platelet inhibition compared to clopidogrel and prasugrel. Ticagrelor has a rapid onset and offset of action, which can be advantageous in clinical situations where quick reversal of antiplatelet effects may be needed [16,17]. Clinical trials have demonstrated that ticagrelor reduces cardiovascular events more effectively than clopidogrel in ACS patients [18]. However, this increased efficacy comes with a higher risk of bleeding, and ticagrelor can cause dyspnoea in some patients [18].

GPIIb/IIIa inhibitors constitute another category of antiplatelet agents. They target the GP IIb/IIIa receptor on platelets, preventing the binding of fibrinogen and vWF, which are essential for platelet aggregation [19]. Abciximab is a monoclonal antibody that irreversibly binds to the GPIIb/IIIa receptor. It has a rapid onset of action and is primarily used during PCI to prevent ischemic complications [20]. Despite its efficacy, abciximab is associated with a high risk of bleeding and thrombocytopenia, which limits its use to high-risk PCI settings where potent platelet inhibition is required [21]. Eptifibatide and tirofiban are small-molecule inhibitors that reversibly bind to the GPIIb/IIIa receptor. These agents have a shorter duration of action compared to abciximab, making them easier to manage in terms of bleeding risk. Both eptifibatide and tirofiban have been shown to reduce ischemic events in patients with ACS and those undergoing PCI. However, the risk of bleeding, though lower than with abciximab, remains a significant concern [22,23].

Recent advancements in the development of novel antiplatelet agents offer promising therapeutic potential for managing ACS (Table 1). Protease-activated receptor (PAR)-1 antagonists target the PAR-1 receptor, which is activated by thrombin, a potent activator of platelets [24]. Vorapaxar is an oral PAR-1 antagonist that inhibits thrombin-induced platelet activation. It has been shown to reduce thrombotic events in patients with a history of myocardial infarction (MI) [25]. However, vorapaxar’s use is limited by an increased risk of bleeding, particularly intracranial haemorrhage, which has led to cautious adoption in clinical practice [25]. Atopaxar is another PAR-1 antagonist that has been investigated in clinical trials. While it has demonstrated efficacy in reducing platelet activation, concerns about bleeding risks, similar to those observed with vorapaxar, have hindered its widespread adoption [26]. The development of safer PAR antagonists with a better balance between efficacy and bleeding risk remains an area of active research [27].

Cangrelol functions as a P2Y12 receptor antagonist with a rapid onset and offset of action. Its effectiveness in inhibiting platelet aggregation has been validated in preclinical studies, and it has successfully completed Phase III clinical trials, gaining FDA approval for clinical application in ACS management [28,29]. Elinogrel, a reversible P2Y12 receptor antagonist, exhibits effective platelet inhibition with reversible effects, demonstrating safety in animal models. It has completed Phase II clinical trials, but further studies are needed to confirm its long-term efficacy and safety [30]. P2Y12-18B, another P2Y12 receptor inhibitor, offers extended duration of action, showing enhanced inhibition of platelet aggregation and a longer half-life compared to clopidogrel. Currently, it has completed Phase I trials and is undergoing Phase II trials. Similarly, Selatogrel, formerly known as ACT-246475, an orally active P2Y12 receptor antagonist, has shown potent platelet inhibition and a favorable pharmacokinetic profile in preclinical models, with ongoing Phase II trials [31]. Selatogrel has the potential for subcutaneous administration and is currently in Phase III trials [32]. BMS-986141, a thromboxane receptor antagonist, reduced thrombus formation and decreased bleeding risk in animal models and has progressed to Phase II trials following successful Phase I trials [33]. RUC-4, a glycoprotein IIb/IIIa receptor antagonist, offers quick inhibition of platelet aggregation, making it potentially useful for emergency settings. It has completed Phase I trials and is planning for Phase II trials [34]. These novel agents, each with unique mechanisms of action, represent significant strides in the development of targeted antiplatelet therapies. Their ongoing clinical trials will determine their efficacy and safety, potentially leading to improved management strategies for ACS and enhanced patient outcomes.

In addition to their antiplatelet effects, aspirin and P2Y12 receptor inhibitors are increasingly recognized for their beneficial anti-inflammatory properties in the context of ACS [35]. Several clinical trials have demonstrated the anti-inflammatory effects of antiplatelet agents in the management of ACS. The CREDO (Clopidogrel for the Reduction of Events During Observation) trial investigated the long-term administration of clopidogrel in 2116 patients undergoing PCI. The results indicated that clopidogrel significantly reduced cardiovascular events with a hazard ratio (HR) of 0.78 (95% confidence interval [CI]: 0.67–0.91; *p* = 0.0015) and lowered inflammatory markers such as C-reactive protein (CRP), highlighting its anti-inflammatory properties in addition to its antiplatelet activity [36]. The CHARISMA (Clopidogrel for High Atherothrombotic Risk and Ischemic Stabilization, Management, and Avoidance) trial compared the combination of clopidogrel and aspirin to aspirin alone in 15,603 patients at high risk for atherothrombotic events. This study found that the combination therapy not only reduced the incidence of MI, stroke, and cardiovascular death (HR: 0.88, 95% CI: 0.77–0.998; *p* = 0.046) but also decreased inflammatory markers, further supporting the anti-inflammatory benefits of clopidogrel [37]. Likewise, findings from the CURE (Clopidogrel in Unstable Angina to Prevent Recurrent Events) inflammatory marker sub-study also showed that measurement of baseline CRP provides supplementary prognostic benefit to standard risk evaluation. Of note, clopidogrel treatment was related to a superior risk reduction than placebo, irrespective of baseline CRP level [38]. Finally, the REDUCE-MVI (Evaluation of Microvascular Injury in Revascularized Patients with ST-Segment–Elevation Myocardial Infarction Treated with Ticagrelor Versus Prasugrel) trial investigated the effect of reduced myocardial infarct size by minimizing microvascular obstruction during percutaneous coronary intervention. The findings demonstrated that intensive antiplatelet therapy significantly reduced infarct size by 15% (*p* < 0.01) and improved microvascular function, suggesting an enhanced anti-inflammatory effect [39]. These trials collectively underscore the significant role of antiplatelet agents in reducing inflammation and improving clinical outcomes in ACS.

Elevated levels of pro-inflammatory CD14^high^CD16^+^ monocytes are associated with ACS, and numerous studies have linked increased CD16^+^ monocyte levels to coronary disease [40]. CD16^+^ monocyte counts are higher in patients with UA compared to those with stable coronary artery disease. Among UA patients, those at intermediate to high risk of MI had significantly higher CD14^high^CD16^+^ monocyte counts [41]. Tapp et al. found a correlation between CD14^high^CD16^+^ counts and both peak troponin-T levels and left ventricular ejection fraction post-STEMI. A study involving 951 patients undergoing elective coronary angiography indicated that higher CD14^high^CD16^+^ counts predict cardiovascular events such as MI, ischemic stroke, and cardiovascular death [42]. Finally, aspirin and clopidogrel have been shown to exert immunomodulatory effects, counteracting the increase in CD14^high^CD16^+^ monocytes under pro-inflammatory conditions. This suggests that these drugs may help reduce monocytic biomarkers of inflammation in cardiovascular disease patients, potentially improving outcomes in ACS [43].

**Table 1:** A concise overview of the novel antiplatelet agents highlighting their mechanisms, preclinical efficacy, and status in clinical trials.

Agent Name	Mechanism of Action	Preclinical Efficacy	Clinical Trials Phase
Vorapaxar	PAR-1 antagonist	Reduced platelet aggregation and thrombus formation in animal models	Phase III completed [25]; approved by FDA
Cangrelor	P2Y12 receptor antagonist	Rapid onset and offset of action, effective inhibition of platelet aggregation	Phase III completed [28]; approved by FDA
Elinogrel	Reversible P2Y12 receptor antagonist	Effective platelet inhibition, reversible effects, and safety in animal models	Phase II completed [30]; further studies needed
Selatogrel (ACT-246475)	Selective P2Y12 receptor antagonist	Rapid and potent platelet inhibition, potential for subcutaneous administration	Phase II completed [31]; Phase III ongoing
BMS-986141	Thromboxane receptor antagonist	Reduced thrombus formation, decreased bleeding risk in animal models	Phase I completed [33]; Phase II ongoing
RUC-4	Glycoprotein IIb/IIIa receptor antagonist	Quick inhibition of platelet aggregation, potential for emergency use	Phase I completed [34], Phase II ongoing

FDA: Food and Drug Administration; PAR-1: Protease-Activated Receptor-1; P2Y12: A subtype of ADP (adenosine diphosphate) receptor found on platelets.

## 3. Anti-Inflammatory Drugs

The emerging role of inflammation in the pathogenesis of ACS has collected increasing attention in recent years, highlighting the complex interplay between inflammatory processes and atherosclerosis. Inflammatory cytokines, such as interleukin-1 (IL-1), interleukin-6 (IL-6), and tumor necrosis factor-alpha (TNF-α), play a pivotal role in orchestrating the inflammatory response in ACS. These cytokines are produced by various cells within the arterial wall, including endothelial cells, macrophages, and smooth muscle cells, in response to vascular injury and atherosclerotic plaque destabilization [44]. Elevated levels of inflammatory cytokines have been observed in ACS patients and are associated with adverse outcomes, including recurrent cardiovascular events and mortality [45].

Leukocytes, including neutrophils, monocytes, and lymphocytes, are key mediators of the inflammatory response in ACS. Upon activation, leukocytes adhere to the endothelium, migrate into the subendothelial space, and release pro-inflammatory cytokines and proteolytic enzymes, promoting plaque destabilization and thrombosis [46]. Neutrophils contribute to endothelial dysfunction and oxidative stress, while monocytes differentiate into macrophages and engulf oxidized low-density lipoprotein (LDL) cholesterol, leading to the formation of lipid-laden foam cells within atherosclerotic plaques [47].

Endothelial dysfunction, characterized by impaired endothelium-dependent vasodilation and increased endothelial permeability, is a hallmark of ACS pathophysiology. Inflammatory mediators, such as cytokines and reactive oxygen species (ROS), disrupt endothelial homeostasis and promote endothelial activation and dysfunction [48]. Endothelial dysfunction contributes to vasoconstriction, platelet activation, and leukocyte recruitment, exacerbating plaque instability and thrombosis [49].

This complex inflammatory cascade plays a critical role in all stages of ACS development, from the initiation and progression of atherosclerosis to plaque rupture and thrombosis. Chronic inflammation promotes the formation of vulnerable plaques characterized by thin fibrous caps, lipid-rich cores, and inflammatory cell infiltration, rendering them susceptible to rupture. Plaque rupture exposes thrombogenic material to the bloodstream, triggering platelet activation and thrombus formation, culminating in acute myocardial ischemia and ACS presentation [50].

Targeting inflammation represents a promising therapeutic strategy for ACS management [51]. Anti-inflammatory agents, such as IL-1β inhibitors and colchicine, have shown significant potential in reducing inflammatory markers and cardiovascular events in ACS patients. Canakinumab, an IL-1β inhibitor, has been extensively studied for its role in mitigating inflammation in cardiovascular diseases [52]. The canakinumab anti-inflammatory thrombosis outcome study (CANTOS) showed that by blocking IL-1β, a key pro-inflammatory cytokine, canakinumab effectively reduces systemic inflammation, thereby lowering the risk of recurrent cardiovascular events. Canakinumab can lead to substantial reductions in high-sensitivity CRP (hs-CRP), a marker of inflammation, and subsequently decrease the incidence of major adverse cardiovascular events (MACE) in patients with a history of MI [53]. While the trial demonstrated that canakinumab significantly reduced the risk of MACE, it also highlighted several side effects. Patients frequently reported infections, including upper respiratory tract infections and more severe infections like pneumonia, bronchitis, and cellulitis. Injection site reactions such as redness, swelling, pain, and itching were common. Gastrointestinal issues like nausea and diarrhea, along with musculoskeletal pain, were also noted. Thus, while canakinumab provides cardiovascular benefits, these must be carefully weighed against the potential risks of serious infections and other side effects [54]. Similarly, colchicine, traditionally used for gout, has emerged as an effective anti-inflammatory agent in the context of cardiovascular disease. It inhibits microtubule formation, which in turn suppresses inflammatory cell activity and cytokine production, contributing to reduced cardiovascular events and improved outcomes in ACS patients [55,56,57]. The LoDoCo2 (Low-Dose Colchicine 2) trial and the COLCOT (Colchicine Cardiovascular Outcomes Trial) are significant studies exploring the cardiovascular benefits of colchicine. LoDoCo2 investigated whether low-dose colchicine could reduce cardiovascular events in patients with chronic coronary disease, demonstrating promising results by showing a significant reduction in cardiovascular events compared to placebo [57]. Meanwhile, COLCOT focused on patients who recently experienced a heart attack and found that colchicine treatment led to a lower risk of cardiovascular events, underscoring its potential in secondary prevention strategies [58]. The most commonly reported side effects in both trials were gastrointestinal disturbances, including diarrhea, nausea, vomiting, and abdominal pain [59]. Both trials highlight colchicine’s role beyond its traditional use, suggesting it may play a pivotal role in managing cardiovascular health by mitigating inflammation and reducing adverse events in at-risk patient populations.

Statins, widely known for their lipid-lowering capabilities, have also been recognized for their pleiotropic effects, including notable anti-inflammatory properties [60]. These effects are particularly beneficial in the prevention and treatment of ACS. Statins inhibit the enzyme HMG-CoA reductase, leading to decreased cholesterol synthesis and upregulation of LDL receptors [61]. Beyond their lipid-lowering effects, statins modulate inflammatory pathways by reducing the levels of pro-inflammatory cytokines, inhibiting the expression of adhesion molecules on endothelial cells, and enhancing the stability of atherosclerotic plaques. This anti-inflammatory action contributes to a reduction in hs-CRP levels, mirroring the benefits observed with direct anti-inflammatory agents [62]. Furthermore, statins have been shown to improve endothelial function, decrease oxidative stress, and reduce thrombogenicity, all of which are critical in mitigating the progression of atherosclerosis and preventing acute coronary events. The dual action of statins, encompassing both lipid-lowering and anti-inflammatory effects, underscores their importance in comprehensive ACS management [63].

Several trials have demonstrated the anti-inflammatory effects of statins in addition to their lipid-lowering properties, particularly in the context of atherogenesis (Table 2). The JUPITER (Justification for the Use of Statins in Primary Prevention: An Intervention Trial Evaluating Rosuvastatin) trial showed that rosuvastatin significantly reduced the risk of cardiovascular events in patients with elevated CRP with a HR of 0.56 and a 95% CI of 0.46–0.69 [64]. The PROVE-IT TIMI 22 (Pravastatin or Atorvastatin Evaluation and Infection Therapy—Thrombolysis in Myocardial Infarction 22) trial compared the effects of intensive atorvastatin therapy with standard pravastatin therapy in patients with acute coronary syndromes. The trial found that intensive statin therapy with atorvastatin led to a greater reduction in cardiovascular events compared to pravastatin, with an HR for death or major cardiovascular events of 0.84 and a 95% CI of 0.70–1.00. This supported the hypothesis that intensive statin therapy provided additional benefits in reducing cardiovascular events, suggesting an anti-inflammatory role for statins [65]. The REVERSAL (Reversal of Atherosclerosis with Aggressive Lipid Lowering trial) assessed the effect of intensive lipid-lowering therapy with atorvastatin compared to moderate therapy with pravastatin on atherosclerosis progression. The results demonstrated that intensive atorvastatin therapy significantly slowed the progression of atherosclerosis, showing a 0.4% reduction in atherosclerosis progression compared to a 2.7% progression in the pravastatin group, with a *p*-value of less than 0.001. This indicated that atorvastatin’s ability to slow atherosclerosis progression further supports its anti-inflammatory properties [66]. The PRINCE (Pravastatin Inflammation/CRP Evaluation) trial was designed to evaluate the effect of pravastatin on CRP levels, an inflammatory marker, in patients with coronary artery disease. The trial found that pravastatin significantly reduced CRP levels, demonstrating its anti-inflammatory effects. This reduction in CRP levels was associated with improved clinical outcomes, supporting the role of statins in reducing inflammation in atherosclerosis [67,68]. In summary, these trials collectively highlight the anti-inflammatory effects of statins in addition to their lipid-lowering properties.

## 4. Agents Targeting Plaque Stabilization

ACS is primarily triggered by the rupture or erosion of vulnerable atherosclerotic plaques, leading to thrombus formation and subsequent myocardial ischemia [69]. Plaque stabilization is critical in preventing these events. Vulnerable plaques, characterized by a large lipid core, thin fibrous cap, and high inflammatory cell content, are prone to rupture [70]. The rupture of these plaques exposes the thrombogenic core to the bloodstream, promoting the formation of a thrombus that can occlude the coronary artery. Stabilizing these plaques by reducing their lipid content, strengthening the fibrous cap, and decreasing inflammation can significantly reduce the risk of rupture and thrombosis, thereby preventing ACS and improving cardiovascular outcomes [71]. Recent research has focused on several novel therapeutic targets (Table 3) aimed at promoting plaque stabilization, particularly by influencing lipid metabolism, extracellular matrix (ECM) remodelling, and vascular smooth muscle cell (VSMC) function [72].

Proprotein convertase cubtilisin/cexin type 9 (PCSK9) inhibitors are novel agents and can lower LDL cholesterol levels by enhancing the clearance of LDL particles from the bloodstream [73]. Clinical trials have shown that PCSK9 inhibitors not only reduce LDL levels but also lower the incidence of cardiovascular events, suggesting a role in plaque stabilization. The FOURIER (Further Cardiovascular Outcomes Research with PCSK9 Inhibition in Subjects with Elevated Risk) trial was a large, randomized, double-blind, placebo-controlled trial that evaluated the efficacy and safety of the PCSK9 inhibitor evolocumab in patients with clinically evident atherosclerotic cardiovascular disease who were on optimal statin therapy [74]. Evolocumab significantly reduced LDL cholesterol levels by about 59% from a median baseline of 92 mg/dL to 30 mg/dL. The most common side effects included nasopharyngitis, upper respiratory tract infections, influenza, back pain, and injection site reactions [75]. Importantly, the trial also demonstrated a significant reduction in the risk of major cardiovascular events. Patients treated with evolocumab experienced a 15% reduction in the composite primary endpoint of cardiovascular death, MI, stroke, hospitalization for unstable angina, or coronary revascularization compared to those receiving placebo [76].

The ODYSSEY OUTCOMES (Evaluation of Cardiovascular Outcomes After an Acute Coronary Syndrome During Treatment with Alirocumab) trial was another large, randomized, double-blind, placebo-controlled trial that investigated the effects of the PCSK9 inhibitor alirocumab in patients who had experienced an ACS within the previous 1 to 12 months and were on high-intensity or maximum-tolerated statin therapy [77]. Alirocumab significantly reduced LDL cholesterol levels by an average of 54.7% compared to placebo. The most common adverse effects were injection site reactions, myalgia or muscle related, upper respiratory tract infection, and nasopharyngitis [78]. The trial demonstrated a significant reduction in the composite primary endpoint of MACE, which included coronary heart disease death, nonfatal MI, fatal and nonfatal ischemic stroke, or unstable angina requiring hospitalization. Patients treated with alirocumab showed a 15% reduction in the risk of MACE compared to those on placebo. Furthermore, there was a notable 15% reduction in all-cause mortality in the alirocumab group [79].

Inclisiran is a small interfering RNA (siRNA) therapy that targets the hepatic synthesis of PCSK9 [80]. This new approach differs from monoclonal antibodies like Evolocumab and Alirocumab, providing an innovative option for lipid management. By silencing the PCSK9 gene, inclisiran reduces the production of PCSK9 protein, leading to increased recycling of LDL receptors on hepatocytes. This results in enhanced clearance of LDL from the bloodstream, thereby significantly lowering LDL levels. The pivotal Phase III clinical trials ORION-9, ORION-10, and ORION-11 were critical in demonstrating the efficacy and safety of inclisiran [81,82]. Olemia andes included patients with atherosclerotic cardiovascular disease (ASCVD) or ASCVD risk equivalents, such as familial hypercholesterolemia, and showed that inclisiran administered biannually significantly reduced LDL-C levels by approximately 50% compared to placebo. The studies also reported a favorable safety profile and high patient adherence due to the infrequent dosing schedule. Inclisiran is generally well-tolerated, but some patients may experience side effects like injection site reactions, nasopharyngitis, headache, back pain, and diarrhea [83].

Apolipoprotein A-I (ApoA-I), the main protein component of high-density lipoprotein (HDL), plays a crucial role in promoting cholesterol efflux and reverse cholesterol transport, which are vital processes in maintaining cardiovascular health [84]. ApoA-I Milano is a variant of this protein that has been shown to enhance these processes, leading to reduced plaque size and improved plaque stability in preclinical animal models [85]. Clinical trials have demonstrated its potential benefits, with Phase II studies showing promising results. The side effects of the drug are rare and include headache, renal impairment, hepatic impairment, and nausea, vomiting, and abdominal pain [86]. The agent’s ability to mimic HDL’s protective functions makes it a significant candidate in the ongoing search for effective plaque-stabilizing therapies [87].

Torcetrapib is a cholesteryl ester transfer protein (CETP) inhibitor developed with the goal of raising HDL cholesterol levels and reducing LDL cholesterol levels. It was hoped that by increasing HDL, and decreasing LDL, it could significantly reduce the risk of cardiovascular events [88]. Initial preclinical studies demonstrated that Torcetrapib significantly increased HDL levels and reduced atherosclerotic lesion size in animal models, suggesting potential benefits in reducing cardiovascular risk. However, despite promising preclinical data, the clinical outcomes were not favourable [89]. The ILLUMINATE trial, a large Phase III study involving Torcetrapib, was terminated prematurely due to safety concerns. While Torcetrapib effectively raised HDL levels and lowered LDL levels, it was associated with a significant increase in all-cause mortality and cardiovascular events [90]. One of the main reasons for the failure of Torcetrapib was its off-target effects, particularly an increase in blood pressure. The hypertensive side-effect did not appear to be mechanism related, but this hypertensive effect counteracted the potential cardiovascular benefits of raising HDL levels. Additionally, Torcetrapib was linked to an increase in aldosterone levels, contributing to its adverse cardiovascular effects [91].

Matrix metalloproteinases (MMPs) are a family of enzymes that play a crucial role in the degradation of extracellular matrix (ECM) components, including collagen and elastin. In the context of atherosclerosis, MMPs contribute to the weakening of the fibrous cap overlying atherosclerotic plaques, thereby promoting plaque instability and increasing the risk of rupture, which can lead to ACS such as MI [92]. Given their critical role in plaque destabilization, MMPs have emerged as a promising therapeutic target for enhancing plaque stability and reducing the incidence of cardiovascular events [93]. Inhibiting MMP activity could potentially strengthen the fibrous cap, preventing plaque rupture and its catastrophic cardiovascular consequences [94].

Several clinical trials and preclinical studies have explored the potential of MMP inhibitors in cardiovascular disease. The MIDAS (Metalloproteinase Inhibition with Subantimicrobial Doses of Doxycycline to Prevent Acute Coronary Syndromes) trial evaluated the effects of doxycycline, a nonselective MMP inhibitor, in patients with coronary artery disease. The study found that doxycycline reduced the levels of MMP-9, a specific MMP implicated in plaque rupture, and improved markers of vascular inflammation and stability [95]. Conversely, doxycycline treatment in two randomized, double-blind, and placebo-controlled clinical trials in individuals with symptomatic coronary and carotid artery disease failed to have any positive result on plaque phenotype or stronger clinical outcome [96,97]. Further large-scale, long-term trials are necessary to conclusively establish the clinical efficacy and safety of MMP inhibitors in preventing cardiovascular events and improving patient outcomes.

Tissue inhibitors of metalloproteinases (TIMPs) are natural inhibitors of MMPs [98]. By inhibiting MMP activity, TIMPs play a crucial role in maintaining ECM integrity and preventing excessive matrix degradation. In the context of atherosclerosis, TIMPs contribute to the stabilization of the fibrous cap covering atherosclerotic plaques, thereby reducing the risk of plaque rupture and subsequent adverse cardiac events [99]. Improving TIMP activity through therapeutic strategies could therefore offer a promising approach to improving plaque stability and reducing the incidence of cardiovascular events. Several preclinical and clinical studies have investigated the potential of TIMPs in cardiovascular disease. Lahdentausta et al. investigate the balance between MMP-9 and endogenous inhibitor, tissue inhibitors of matrix metalloproteinase 1 (TIMP-1), in ACS patients. They found that MMP-9 and the MMP-9/TIMP-1 molar ratio may be valuable in ACS diagnosis and prognosis. High serum MMP-9 activation potential is associated with poor cardiovascular outcome [100]. Johson et al. demonstrated that overexpression of TIMP-1 in atherosclerotic mice led to reduced MMP activity, decreased plaque vulnerability, and increased fibrous cap thickness, highlighting the role of TIMPs in plaque stabilization [101]. These findings underscore the potential of MMPs and TIMPs enhancement as a therapeutic strategy for stabilizing atherosclerotic plaques and preventing ACS events. Further research and clinical trials are needed to fully elucidate the therapeutic potential of TIMPs in cardiovascular disease and to develop targeted treatments that leverage their protective effects.

The MCP-1 inhibitor targets the monocyte chemoattractant protein-1 (MCP-1), a key chemokine involved in the recruitment of monocytes to sites of inflammation, such as atherosclerotic plaques [102]. By inhibiting MCP-1, this agent reduces monocyte infiltration into plaques, thereby decreasing inflammation and stabilizing the plaques [103]. Preclinical models have shown that MCP-1 inhibitors can effectively reduce macrophage content in plaques and enhance plaque stability. Ongoing Phase II clinical trials are evaluating the efficacy and safety of MCP-1 inhibitors in patients with atherosclerotic cardiovascular disease [104].

D-4F is an apolipoprotein A-I (ApoA-I) mimetic peptide designed to enhance cholesterol efflux and reverse cholesterol transport, processes critical for maintaining cardiovascular health [105]. By mimicking ApoA-I, D-4F promotes the removal of cholesterol from atherosclerotic plaques and reduces oxidative stress and inflammation. Preclinical studies have demonstrated that D-4F can significantly reduce atherosclerosis and stabilize plaques in animal models [106,107]. Clinical trials have progressed through Phase I/II, showing promising results in reducing atherosclerotic burden and improving lipid profiles [108].

AZD5718 is an inhibitor of 5-lipoxygenase activating protein (FLAP), which plays a key role in the biosynthesis of leukotrienes, inflammatory mediators involved in the pathogenesis of atherosclerosis [109]. By inhibiting FLAP, AZD5718 reduces leukotriene production, thereby decreasing inflammation and stabilizing atherosclerotic plaques. Preclinical studies have shown that AZD5718 effectively reduces plaque inflammation and size [110]. No serious or adverse effects have been monitored so far. Phase II clinical trials are ongoing to evaluate the drug’s efficacy and safety in patients with cardiovascular disease [111].

Darapladib is an inhibitor of lipoprotein-associated phospholipase A2 (Lp-PLA2), an enzyme implicated in the inflammatory processes of atherosclerosis [112]. By inhibiting Lp-PLA2, Darapladib reduces inflammation and necrosis within atherosclerotic plaques. Darapaglid adverse effects include asthma, anaphylaxis, diarrhea, and unpleasant odor of skin, urine, or feces [113]. Preclinical studies showed promising results with decreased plaque necrosis and inflammation [114]. However, Phase III clinical trials did not demonstrate a significant reduction in MACEs, leading to the conclusion that while Darapladib can reduce plaque inflammation, it does not significantly impact overall cardiovascular outcomes [115,116].

Finally, we should mention the angiotensin-converting enzyme (ACE) inhibitors and the angiotensin II receptor blockers (ARBs), as these agents, together with their anti-hypertensive action, play a crucial role in stabilizing atherosclerotic plaques by modulating the renin-angiotensin-aldosterone system (RAAS) [117]. These agents effectively reduce VSMC proliferation and migration, key processes in plaque development and instability. Additionally, they decrease inflammation within the arterial wall, contributing to a more stable plaque phenotype. Several studies have shown that these agents effectively decrease VSMC activity, contributing to a more stable plaque phenotype. The HOPE (Heart Outcomes Prevention Evaluation) trial demonstrated that the ACE inhibitor ramipril significantly reduced the incidence of MI, stroke, and cardiovascular death in patients at high risk for cardiovascular events [118]. Similarly, the LIFE (Losartan Intervention for Endpoint Reduction in Hypertension) study found that the ARB losartan reduced the risk of stroke compared to atenolol in hypertensive patients with left ventricular hypertrophy [119]. Additionally, ACE inhibitors and ARBs have been shown to reduce markers of inflammation, such as CRP and IL-6, further supporting their role in stabilizing atherosclerotic plaques. These findings highlight the multifaceted benefits of RAAS inhibition in managing atherosclerosis and preventing acute coronary syndromes by promoting plaque stability through the modulation of VSMC behaviour and inflammatory processes.

**Table 3:** Overview of the promising novel agents targeting plaque stabilization, summarizing their mechanisms of action, efficacy in preclinical studies, and the status of their clinical trials.

Agent Name	Mechanism of Action	Preclinical Efficacy	Clinical Trials Phase
ApoA-I Milano	HDL Mimetic, promotes cholesterol efflux	Reduced plaque size and improved plaque stability in animal models	Phase II completed [85]
Evolocumab	PCSK9 Inhibitor, reduces LDL levels	Significant reduction in atherosclerotic plaque burden in preclinical studies	Phase III completed [76]; FDA approved
Alirocumab	PCSK9 Inhibitor, reduces LDL levels	Decreased plaque size and improved plaque stability in animal models	Phase III completed [79]; FDA approved
Inclisiran	PCSK9 Inhibitor, reduces LDL levels	Significantly reduced LDL-C levels by approximately 50% compared to placebo.	Phase III completed [81,82]; FDA approved
Torcetrapib	CETP Inhibitor, increases HDL levels	Decreased vascular inflammation and reduced plaque progression in animal models	Discontinued in Phase III due to side effects [90]
Doxycycline	MMP Inhibitor, reduces matrix metalloproteinase activity	Reduced plaque instability and inflammation in animal models	Phase II completed [95]
MCP-1 Inhibitor	Chemokine Receptor Antagonist, reduces monocyte recruitment	Reduced macrophage infiltration and stabilized plaques in preclinical models	Phase II ongoing [104]
D-4F	ApoA-I Mimetic Peptide, promotes cholesterol efflux	Reduced atherosclerosis and stabilized plaques in animal studies	Phase I/II completed [108]
AZD5718	FLAP Inhibitor, reduces leukotriene production	Reduced plaque inflammation and size in preclinical studies	Phase II ongoing [111]
Darapladib	Lp-PLA2 Inhibitor, reduces inflammation	Decreased plaque necrosis and inflammation in animal models	Phase III completed [115,116], not approved

CETP: Cholesteryl ester transfer protein; FDA: Food and Drug Administration; FLAP: 5-lipoxygenase activating protein; HDL: High-density lipoprotein; LDL: Low-density lipoprotein; Lp-PLA2: Lipoprotein-associated phospholipase A2; MCP-1: Monocyte chemoattractant protein-1; MMP: Matrix metalloproteinase; PCSK9: Proprotein convertase subtilisin/kexin type 9.

## 5. Integration of Novel Therapeutic Strategies Into Current Clinical Practice

The current guidelines for managing ACS are well established and emphasize prompt reperfusion therapy, DAPT, statin therapy, and secondary prevention measures. These guidelines, such as those outlined by the European Society of Cardiology (ESC) [120] and the American College of Cardiology (ACC) [121], focus on rapid diagnosis and immediate management strategies followed by long-term management with antiplatelet agents, beta-blockers, ACE inhibitors, and lifestyle modifications. The recent advancements in ACS therapeutics, as discussed earlier, suggest novel approaches that aim to target different pathways involved in the pathophysiology of ACS. Integrating these emerging therapies into clinical practice and clinical guidelines presents both opportunities and challenges. For example, the inclusion of anti-inflammatory agents like canakinumab and colchicine, which have demonstrated efficacy in reducing inflammatory markers and cardiovascular events, could complement existing treatments and provide additional benefits in managing residual inflammatory risk in ACS patients. Similarly, PCSK9 inhibitors offer a novel mechanism of action by enhancing LDL clearance, which could be particularly beneficial for patients with hypercholesterolemia and those who are statin-intolerant.

The current European guidelines reinforce the use of PCSK9 inhibitors during ACS hospitalization in patients who were not at their LDL-C goal despite being on statin and ezetimibe treatment before admission (Class 1, Level A) [120]. The guidelines also support the use of low-dose colchicine in patients with acute coronary syndromes and insufficiently controlled cardiovascular risk factors (Class IIb, Level A) [120]. However, more evidence is needed for other promising therapeutic targets to integrate into the guidelines. Overall, while the current guidelines provide a robust framework for ACS management, incorporating emerging therapeutic strategies could further enhance patient outcomes. Future research and clinical trials will be crucial in validating the efficacy and safety of these novel agents, facilitating their integration into routine clinical practice, and ultimately advancing the standard of care for ACS patients.

## 6. Challenges and Unmet Needs

One of the primary challenges in translating preclinical findings into clinical practice for emerging therapeutic targets in ACS is the potential for off-target effects [122]. Many novel therapeutic agents target specific molecular pathways or cellular processes implicated in ACS pathophysiology. However, these interventions may also interact with unintended targets, leading to off-target effects that can manifest as adverse reactions or unexpected outcomes in clinical trials. Identifying and mitigating off-target effects through rigorous preclinical testing, including in vitro studies and animal models, is essential to minimize the risk of adverse events and optimize the safety profile of novel therapies before advancing to clinical trials [123].

Patient heterogeneity presents another significant challenge in translating preclinical findings into clinical practice for ACS [124,125]. Preclinical studies often involve homogeneous animal models or cell cultures with controlled experimental conditions, whereas clinical trial populations are inherently heterogeneous, reflecting the diversity of patients encountered in real-world clinical practice [126]. Factors such as age, sex, comorbidities, genetic background, and concomitant medications can influence treatment responses and complicate the interpretation of clinical trial results. Addressing patient heterogeneity through appropriate patient selection criteria, subgroup analyses, and personalized medicine approaches is crucial to ensure the generalizability and applicability of trial findings to diverse patient populations with ACS [127].

Designing clinical trials that effectively evaluate the safety and efficacy of emerging therapeutic targets for ACS presents several challenges [128]. Issues such as selection bias, randomization procedures, blinding techniques, and outcome assessment methods must be carefully considered to minimize bias and ensure the validity and reliability of trial results. Promising future research direction might offer the development of heterogeneous treatment effects (HTE) estimation methods that influence the similar mechanism of treatment and placebo, together with an effort to complement the incomplete healthcare data incorporating temporal relationships of the variables when estimating HTE [129]. Evaluating HTE with machine learning methods presents internal challenges since most machine learning algorithms are optimized for prediction rather than for estimating causal effects [130]. Finally, determining appropriate endpoints that accurately reflect clinically meaningful outcomes, such as cardiovascular events, mortality, and quality of life, is essential for evaluating the therapeutic benefit of novel interventions in clinical practice. Following the model of biological mechanism-driven therapy, the new biomarker-guided clinical trial designs including basket, umbrella, and platform trials have successfully transformed these possibilities into clinical benefits for patients [131]. Collaborating with multidisciplinary teams, including clinicians, statisticians, trial-related researchers and regulatory experts, is critical to designing robust clinical trials that address the specific challenges associated with translating preclinical findings into clinical practice for ACS [131].

In conclusion, translating promising preclinical findings into clinically effective therapies for ACS requires careful consideration of the challenges and limitations inherent in the translational process. By addressing issues such as off-target effects, patient heterogeneity, and trial design considerations, researchers and clinicians can enhance the likelihood of successful translation and ultimately improve patient outcomes in clinical practice.

## 7. Future Directions

Future research directions in ACS therapeutics hold the potential for further advancing treatment strategies and improving patient outcomes. At first, combination therapies might be beneficial. By combining multiple therapeutic agents in a polypill, targeting different pathways implicated in ACS pathophysiology may yield synergistic effects and enhance treatment efficacy [132,133]. For example, the investigation of optimal combinations of P2Y12 inhibitors, glycoprotein IIb/IIIa inhibitors, and PAR antagonists to maximize platelet inhibition and reduce thrombotic events. Another area for combined therapy would be the study of potential benefits of combining traditional antiplatelet agents with novel anti-inflammatory drugs (e.g., interleukin-1β inhibitors) to mitigate inflammation-mediated plaque instability and thrombosis. However, potential drug interactions, optimizing dosing regimens, and assessing safety profiles are critical considerations in designing and implementing combination therapies for ACS [134].

Recognizing, however, the heterogeneity of ACS patients and their varying responses to treatment underscores the importance of personalized medicine tailored to individual patient characteristics and underlying pathophysiology [135]. For example, genetic profiling can identify variants associated with differential responses to antiplatelet therapy, allowing for tailored treatment strategies [136]. Biomarker-guided therapy, utilizing markers like high-sensitivity C-reactive protein (hs-CRP) and troponin levels, can guide treatment decisions and risk stratification in ACS patients [137]. However, challenges include the logistical and regulatory barriers, the integration of genetic testing into routine clinical practice, and the establishment of robust algorithms for biomarker-driven decision making [138].

**Figure 1:**
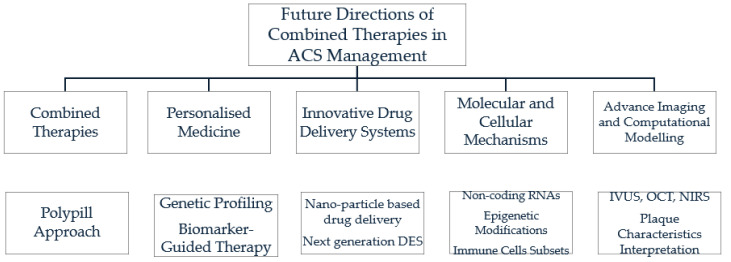
Future directions of combined therapies in acute coronary syndrome management. ACS: Acute coronary syndrome; DES: Drug Eluting stent; RNAs: Ribonucleic Acids; IVUS: Intravascular ultrasound; OCT: Optical coherence tomography; NIRS: Near-infrared spectroscopy.

Innovative drug delivery systems offer the potential to enhance drug efficacy, improve patient adherence, and minimize off-target effects in ACS therapy. Examples include nanoparticle-based drug delivery, which targets atherosclerotic plaques for site-specific drug release [139], and next-generation drug-eluting stents (DES) with improved biocompatibility, controlled drug release kinetics, and enhanced anti-restenotic properties for PCI in ACS patients [140,141]. Furthermore, expanding our understanding of the underlying molecular and cellular mechanisms driving ACS pathophysiology is essential for identifying novel therapeutic targets and optimizing treatment strategies. For instance, investigating the role of non-coding RNAs [142], epigenetic modifications [143], and immune cell subsets in ACS pathogenesis and progression is critical [144]. Finally, utilizing advanced imaging modalities (e.g., intravascular ultrasound, optical coherence tomography) and computational modeling techniques can help interpret plaque characteristics and predict clinical outcomes. In conclusion, future research in ACS therapeutics should focus on exploring combination therapies, personalized medicine approaches, and novel drug delivery systems to address the multifactorial nature of ACS and improve patient outcomes (Figure 1).

## 8. Conclusions

The integration of anti-inflammatory strategies, including the use of IL-1β inhibitors, colchicine, statins, PCSK9 inhibitors, and other novel treatments represents a multifaceted approach to ACS management. By addressing lipid abnormalities, plaque stabilization, and the underlying inflammatory processes, these therapies may offer a synergistic benefit, improving patient outcomes. The evidence supporting the use of anti-platelet agents, anti-inflammatory agents, and statins in reducing cardiovascular events highlights the evolving landscape of ACS treatment. Future research is expected to further delineate the mechanisms through which these therapies exert their beneficial effects and to identify optimal strategies for their use in clinical practice. As the understanding of inflammation in cardiovascular disease expands, the incorporation of targeted anti-inflammatory therapies alongside traditional treatments holds promise for more effective and personalized management of ACS, ultimately reducing the burden of this prevalent and life-threatening condition.

## Figures and Tables

**Table 2:** Summary of major trials evaluating statins as anti-inflammatory agents in atherogenesis.

Name of the Study	Year	Studied Statin	Number of Participants	Disease of the Participants	Results
JUPITER (Justification for the Use of Statins in Prevention: an Intervention Trial Evaluating Rosuvastatin) [64]	2008	Rosuvastatin	17,802	Elevated CRP, normal LDL	HR for major cardiovascular events: 0.56 (95% CI: 0.46–0.69); significant reduction in events
PROVE-IT TIMI 22 (Pravastatin or Atorvastatin Evaluation and Infection Therapy—Thrombolysis in Myocardial Infarction 22) [65]	2004	Atorvastatin	4162	Acute coronary syndromes	HR for death or major cardiovascular events: 0.84 (95% CI: 0.70–1.00); greater reduction in events with intensive therapy
REVERSAL (Reversal of Atherosclerosis with Aggressive Lipid Lowering) [66]	2004	Atorvastatin	502	Coronary artery disease	0.4% reduction in atherosclerosis progression vs. 2.7% progression with pravastatin; *p* < 0.001
PRINCE (Pravastatin Inflammation/CRP Evaluation) [67]	2001	Pravastatin	1702	Coronary artery disease	Significant reduction in CRP levels; improved clinical outcomes

CI: Confidence interval; CRP: C-reactive protein; HR: Hazard ratio; LDL: Low-density lipoprotein; TIMI: Thrombolysis in Myocardial Infarction.

## Data Availability

Data are contained within the article.
